# Examining the association between social media fatigue, cognitive ability, narcissism and misinformation sharing: cross-national evidence from eight countries

**DOI:** 10.1038/s41598-023-42614-z

**Published:** 2023-09-18

**Authors:** Saifuddin Ahmed, Muhammad Ehab Rasul

**Affiliations:** 1 Wee Kim Wee School of Communication and Information, Nanyang Technological University, Singapore, Singapore; 2 Department of Communication, University of California, Davis, Davis, USA

**Keywords:** Diseases, Health care

## Abstract

Several studies have explored the causes and consequences of public engagement with misinformation and, more recently, COVID-19 misinformation. However, there is still a need to understand the mechanisms that cause misinformation propagation on social media. In addition, evidence from non-Western societies remains rare. This study reports on survey evidence from eight countries to examine whether social media fatigue can influence users to believe misinformation, influencing their sharing intentions. Our insights also build on prior cognitive and personality literature by exploring how this mechanism is conditional upon users’ cognitive ability and narcissism traits. The results suggest that social media fatigue can influence false beliefs of misinformation which translates into sharing on social media. We also find that those with high levels of cognitive ability are less likely to believe and share misinformation. However, those with low cognitive ability and high levels of narcissism are most likely to share misinformation on social media due to social media fatigue. This study is one of the first to provide cross-national comparative evidence highlighting the adverse effects of social media fatigue on misinformation propagation and establishing that the relationship is not universal but dependent on both cognitive and dark personality traits of individuals.

## Introduction

Recently, the world has witnessed severe consequences posed by the COVID-19 pandemic. People who were forced to stay home increasingly relied on social media for various purposes, such as news consumption, coping with stress, remote working, and sharing their opinions regarding the pandemic^1–4^. Unfortunately, the increased reliance on social media during COVID-19 gave rise to greater engagement with COVID-19 misinformation^5^ and misinformation-sharing behavior^6^. As such, the World Health Organization and scholars raised concerns about an emerging “infodemic,” wherein attitudes may be influenced by a range of misinformation^7–10^.

The heightened concerns about the role of social media in misinformation spread have spurred an emerging body of research to consider the psychology of COVID-19 misinformation engagement in many societies. The effects of misinformation engagement on social media vary among people, with some more susceptible than others^11^. However, the drivers of public engagement with COVID-19 misinformation are often the same as general political misinformation. Those who are older, low educated, and with lower cognitive ability are more likely to engage and endorse COVID-19 misinformation. Numerous studies have also highlighted social media's role in amplifying the spread of misinformation^2–4^. These findings add to the literature on citizen engagement with misinformation^5–9^. However, it also needs to address several drawbacks addressed in this study.

First, while existing research has examined the link between different types of social media *use* and misinformation engagement, the current study focuses on the consequences of *amplified* social media use on misinformation belief and endorsement in the form of sharing. The mass amount of information available on social media and a large amount of unclear information during COVID-19 led to an information overload for users^12^. Consequently, this may have resulted in social media fatigue (SMF)^13^. SMF may impact misinformation belief and endorsement through sharing on social media. Existing scholarship defines SMF as an impulse to limit social media usage due to information and cognitive overload^13^, an evaluation of perceived tiredness as a result of social media use^14^, and “a temporary, however systematically triggered, state of fatigue caused by social media use”^15^. SMF may lead to increased engagement with misinformation (e.g., belief, endorsement, etc.) due to higher information and cognitive overload. Indeed, some studies found positive relationships between cognitive overload and misinformation sharing^15^. As more and more people turn to social media, these effects are likely to be more pronounced and require scholarly attention.

The cognitive overload theory (CLT) posits that humans have a limited working memory that can be overwhelmed if presented with too much information^16^. During COVID-19, people were exposed to troves of information on social media. In turn, people were likely to have reached their cognitive limit, leaving them too exhausted to handle new information^17^. Also, social media platforms often expose users to irrelevant information that could be more organized and easily understandable, which may also lead to information overload^18^. This information overload can cause SMF due to less systematic processing of information. Scholars have found that a lack of deliberation and careful reasoning negatively impacts the discernment between misinformation and truthful information^19^. When an individual experiences SMF, they are less likely to pay careful attention and filter the information they encounter on social media^20^, increasing their vulnerability to misinformation. While scholars have linked information processing with misinformation engagement, cognitive ability is an important factor in misinformation engagement. Existing literature has maintained that cognitive ability is distinct from analytical thinking, which is a cognitive style of information processing^21^. Studies have found that those with high levels of cognitive ability were likely to fall for misinformation inconsistent with their beliefs^22^. In contrast, other work has shown that individuals high in cognitive ability are less likely to fall for misinformation and believe in the paranormal, but only among those who value epistemic rationality^23^. Some evidence suggests that SMF can also impact misinformation engagement via cognitive ability. Specifically, SMF can lower cognitive ability, leading to lower concentration^24,25^. Subsequently, this can induce information efficacy, which discourages people from critically evaluating information^26^. Moreover, the nature of social media algorithms may have resulted in repeated exposure to misinformation, which can increase trust in false beliefs through the illusory truth effect, which maintains that repeated information is seen as more truthful^27,28^.

As such, we hypothesize:

- **H1a**: SMF will be positively associated with the perceived accuracy of misinformation.
- **H1b**: SMF will be positively associated with the sharing of misinformation.
- **H2**: The relationship between SMF and misinformation sharing will be mediated by the perceived accuracy of misinformation.

Second, existing research has identified that misinformation engagement varies among individuals based on their cognitive ability^5^ and personality traits^29,30^. However, empirical frameworks considering both sets of factors together are limited. In a rare attempt, a recent study examined the conditional influence of cognitive ability and big five personality traits. Though, attention to the confluence of cognitive and dark personality traits is absent. Below, we outline our propositions regarding cognitive ability, dark personality traits (narcissism), and misinformation sharing.

A recent study found that people with higher levels of cognitive ability are less prone to COVID-19 misinformation through memory formation^31^. Similarly, others have found that people with high levels of cognitive ability were less likely to share COVID-19 misinformation on social media^5,32^. People with high levels of cognitive ability also need less strong arguments to accept rebuttals to COVID-19 misinformation, while those with lower cognitive ability require stronger arguments^33^. When exposed to misinformation on social media, those who critically process the information may identify false news more accurately. This can be explained by motivated system two reasoning, a part of the dual-process theory, which posits that the systematic processing of information can outweigh the automatic response to information^34^. Accordingly, people with high cognitive ability levels are more likely to engage in more analytical thinking and be skeptical of misinformation and false news^35^. Therefore, we hypothesize the following:

- **H3a**: Cognitive ability will be negatively associated with the perceived accuracy of misinformation.
- **H3b**: Cognitive ability will be negatively associated with misinformation sharing.

In the present study, we extend the current literature and examine whether narcissism impacts belief and sharing of misinformation. One line of inquiry in this area has suggested that personality traits impact misinformation engagement^30^. For example, extroverted people are more likely to perceive misinformation as accurate and share it, while people high in neuroticism and openness are more likely to perceive it as accurate^5^. Although individual traits play a role in misinformation engagement, the tendency to have extreme variants of personality traits has been linked with compulsive behavior and psychopathy^36,37^. Indeed, traits such as neuroticism have been linked with COVID-19 misinformation and conspiracy theory engagement^38^. Other scholarship has also found that those with the dark triad of personality traits (narcissism, psychopathy, and Machiavellianism) are more likely to share false information on social media^39^. Even though personality traits offer a nuanced understanding of the psychological aspects of COVID-19 misinformation engagement, we argue that narcissism can provide insights into the processes involved in misinformation belief and sharing. A multi-country study found that national narcissism, a form of narcissism, is positively related to COVID-19 conspiracy beliefs^40^. Another recent study found that some forms of narcissism can negatively impact adherence to COVID-19 mitigation policies, such as mask-wearing and vaccination^41^. Narcissism has been identified as a multi-dimensional construct, expanding its utility in understanding the psychological processes underpinning misinformation engagement. For instance, collective narcissism, another form of narcissism, is also linked with belief in COVID-19 conspiracy theories^42^. It must be noted here that collective narcissism is a different construct than narcissism. Narcissism is also characterized by an increased desire for attention, admiration, and feelings of uniqueness^43^. Narcissists may then engage in the sharing of misinformation as it may appear as more privileged information^44^. Prior research has illustrated that narcissists are more active online^45^, increasing their chances of being exposed to misinformation. In fact, one study found that narcissism is associated with belief in misinformation when individuals are repeatedly exposed to misinformation which, in turn, leads to greater sharing of false information^46^. However, some researchers have argued that narcissists’ heightened self-regulatory abilities and hyper-focus can assist them in detecting false news^47^. On the other hand, narcissists grossly overestimate their performances on tasks^48,49^. Subsequently, narcissists may be more prone to misinformation belief, and sharing. Hence, we hypothesize that:

- **H4a**: Narcissism will be positively associated with the perceived accuracy of misinformation.
- **H4b**: Narcissism will be positively associated with misinformation sharing.

While previous research has examined the role of cognitive ability and narcissism in misinformation engagement separately^5,33,35,40–42^, they have not been studied together. Cognitive ability and narcissism may differentially impact misinformation belief and sharing as they are two crucial cognitive and psychological aspects that scholars have widely studied. As such, we propose the following research question:

*RQ1*: How do cognitive ability and narcissism moderate the mediated relationship between SMF and sharing of COVID-19 misinformation through perceived accuracy?

In summary, while the existing scholarship on COVID-19 and misinformation on social media offers interesting insights, there are a few prevalent research gaps. First, most current studies have yet to focus on SMF as a predictor of misinformation during COVID-19 (as an exception, see 20). SMF has been associated with sharing COVID-19 misinformation, a key concern for academics^15^. Second, while scholars have assessed cognitive ability’s impact on social media and misinformation engagement^29,38^, it has not been studied together with narcissism, which is a dark personality trait associated with misinformation engagement^40–42^. It is essential to study cognitive ability and narcissism in conjunction as they can offer important insights into the psychology of misinformation belief and sharing, allowing academics and policymakers to propose strategies to minimize the harmful impact of misinformation.

In addition, most of the existing literature focuses on Western countries such as the US^29,35^ and ignores Asian countries other than China and Singapore^1,5^. The Asian population makes up a large portion of the global social media user base and, therefore, may be exposed to misinformation related to COVID-19. For example, Asia has approximately 2.14 billion active social media users^50^. As a result, people in Asia may have been exposed to large amounts of misinformation on social media during COVID-19, which is challenging since Asian countries have faced some of the worst consequences of COVID-19.

**Figure 1:**
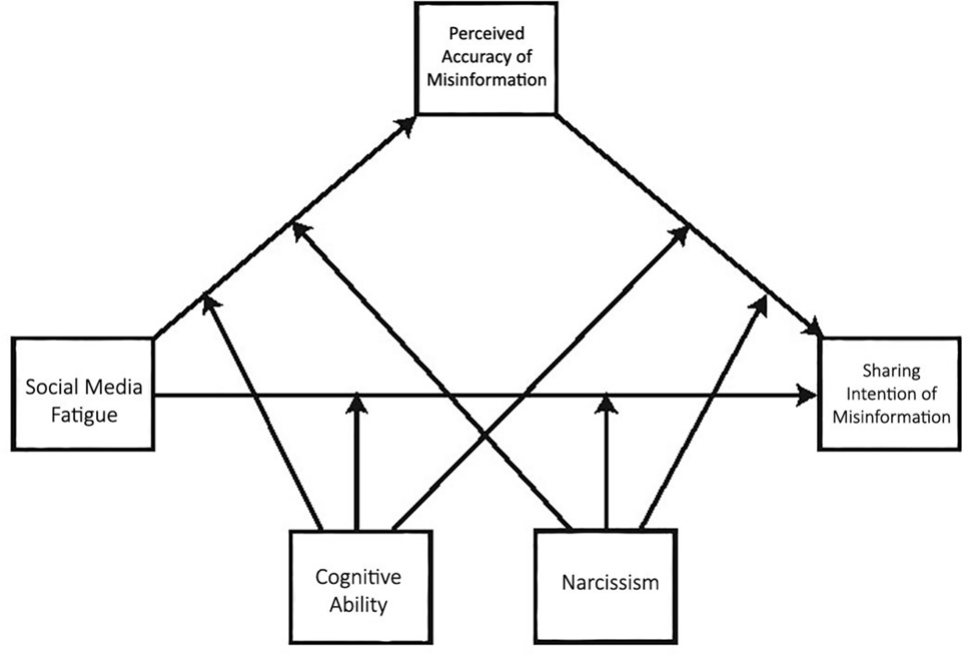
Conceptual framework.

The current study further addresses gaps in the existing literature by proposing a conceptual model which elucidates how SMF impacts misinformation belief and sharing. Specifically, we argue that SMF leads to COVID-19 misinformation sharing through the perceived accuracy of the misinformation (a mediated relationship). Further, we contend that this relationship is moderated by levels of cognitive ability and narcissism (see Fig. 1 for an illustration). We focus on cognitive ability and narcissism as they have been positively linked with COVID-19 misinformation engagement in existing literature^5,33,35,40–42^. Cognitive ability and narcissism offer nuanced insights into the psychological processes involved in misinformation belief and sharing on social media.

In the current study, we rely on survey data from the US, China, Singapore, Indonesia, Malaysia, Philippines, Thailand, and Vietnam. Among these countries, China, Singapore, Indonesia, Malaysia, the Philippines, Thailand, and Vietnam are important and densely populated countries in Asia. Including the US in our dataset allows for comparative analyses that may shed light on important cultural differences in how cognitive ability and narcissism impact COVID-19 misinformation belief and sharing. Also, we rely on a quota sampling strategy based on demographics, which allows our findings to generalize to the larger population.

## Methods

### Sample

Qualtrics LLC administered the surveys for this study, a reputed survey agency often used in social science research. The surveys were launched in June 2022 across eight countries, including the US, China, Singapore, Indonesia, Malaysia, Philippines, Thailand, and Vietnam. The surveys were conducted in the English language in the US and Singapore and were translated into the regional languages of other countries. We utilized quota sampling techniques where the survey sample was matched to the population (age and gender quotas) to increase the representativeness of the findings. Overall, we collected 8070 responses (final response rate = 76%) with US (N = 1010), China (N = 1010), Singapore (N = 1008), Indonesia (N = 1010), Malaysia (N = 1002), Philippines (N = 1010), Thailand (N = 1010), Vietnam (N = 1010). The demographic details for each country in included in Table 1.

**Table 1:** Mean, Standard Deviation of all variables under study.

	US	China	Singapore	Indonesia	Malaysia	Philippines	Thailand	Vietnam
Male	46.0%	51.5%	54.9%	50.2%	49.2%	51.3%	52.9%	53.2%

	Mean (SD)	Mean (SD)	Mean (SD)	Mean (SD)	Mean (SD)	Mean (SD)	Mean (SD)	Mean (SD)
Age	49.0 (17.5)	38.0 (12.7)	43.8 (14.0)	40.3 (13.0)	39.9 (13.7)	37.2 (13.6)	41.6 (13.0)	38.4 (12.8)
Education	5.22 (1.21)	5.72 (.998)	5.32 (1.22)	5.27 (1.06)	5.77 (1.61)	5.38 (1.11)	5.34 (1.17)	5.55 (.943)
Income	5.79 (3.76)	6.00 (2.27)	5.47 (2.64)	2.81 (2.39)	4.14 (2.51)	3.26 (2.02)	4.09 (1.91)	6.52 (2.03)
Political interest	3.11 (1.27)	3.28 (.99)	2.73 (1.08)	2.87 (1.02)	2.93 (1.04)	3.35 (.98)	3.17 (1.07)	3.40 (.97)
SM news	2.90 (1.49)	3.27 (1.22)	3.17 (1.23)	3.49 (1.21)	3.76 (1.21)	4.17 (0.97)	4.21 (1.03)	3.67 (1.07)
TV news	3.35 (1.35)	3.26 (1.17)	3.11 (1.22)	3.13 (1.24)	3.31 (1.23)	3.94 (1.08)	3.68 (1.22)	3.28 (1.09)
Radio news	2.40 (1.28)	2.46 (1.16)	2.44 (1.22)	2.07 (1.09)	2.64 (1.13)	3.07 (1.22)	2.43 (1.19)	2.32 (1.11)
Print news	2.20 (1.29)	2.12 (1.03)	2.51 (1.31)	2.11 (1.15)	2.63 (1.24)	2.59 (1.23)	2.37 (1.11)	2.33 (1.03)
Perceived accuracy	2.14 (1.15)	1.95 (0.87)	1.72 (.90)	2.21 (.080)	2.13 (0.89)	2.48 (.083)	2.26 (0.97)	2.18 (0.92)
Sharing intention	2.01 (1.25)	2.12 (1.06)	1.63 (0.94)	2.23 (1.14)	2.21 (1.11)	2.44 (1.12)	2.05 (1.06)	2.27 (0.92)
SMF	2.61 (1.13)	2.77 (0.94)	2.80 (1.03)	2.73 (0.95)	3.02 (0.87)	2.77 (0.93)	3.00 (0.95)	2.85 (0.97)
Cognitive ability	5.43 (2.41)	8.00 (2.09)	5.53 (2.30)	4.78 (1.19)	4.47 (1.79)	6.56 (2.14)	5.81 (1.67)	5.23 (1.86)
Narcissism	2.67 (1.12)	3.38 (0.83)	2.72 (1.02)	2.62 (0.89)	2.78 (0.97)	2.88 (0.92)	3.20 (0.90)	3.33 (0.93)

SM news, social media news; TV news, television news; SMF, social media fatigue.

All participants provided informed consent prior to taking part in the study. The study was approved by the institutional review board at Nanyang Technological University. Also, all methods were carried out in accordance with the guidelines and regulations.

### Measures

*Social media fatigue* was measured using an existing measure^51^. The participants were asked to rate their level of agreement (1 = strongly disagree to 5 = strongly agree) regarding five items concerning their social media use. Sample items include: “during social media use, I often feel too fatigued to perform other tasks well” and “due to using social media, I feel rather mentally exhausted.” The responses to the five items were averaged to create an index of social media fatigue.

*Perceived accuracy of misinformation* was measured by questioning the participants to rate their perceived accuracy (1 = not at all accurate to 5 = extremely accurate) for COVID-19-related news headlines on social media. The false claims were presented to the participants in a mock social media post style. The misinformation included a) coconut is effective in reducing COVID-19 symptoms, b) COVID-19 vaccinations can cause magnetism by introducing graphene oxide into the blood, c) COVID-19 vaccinations are dangerous and ineffective against Omicron variants, and d) the lifestyle healing program of an alkaline diet, exercise, and healing foods can cure COVID-19. This approach has been previously adopted to measure the perceived accuracy of misinformation^23,35^. Here, the answer to the four questions was averaged to create an index of the perceived accuracy of misinformation.

*Misinformation sharing* was measured by asking the participants to rate their likelihood of sharing (1 = extremely likely to 5 = not at all likely) the news headlines on social media. The values were reverse coded such that a higher value represents greater sharing intention. Finally, the response to the four questions (same as perceived accuracy) was averaged to create an index of misinformation sharing.

It can be argued that this approach captures sharing intentions, not real-world behavior. Still, this approach has been frequently adopted to measure sharing behavior^52,53^. Also, self-reports of such sharing intentions are often found to be strongly related to real-world attention to news headlines^54^. Therefore, while not capturing real-world sharing, our approach would shed light into sharing behavior of social media users.

*Cognitive ability* was measured by the wordsum test. The test includes ten questions where participants are given a source word (e.g., ‘emanate’) and are required the match the word with the closest associated word from a list of five target words (e.g., ‘populate,’ ‘free,’ ‘prominent,’ ‘rival,’ ‘come’). The wordsum test is strongly associated with general measures of intelligence and has been frequently employed in previous studies to measure the cognitive ability of participants^55,56^. The test has also been used in studies examining the association between cognitive ability and misinformation engagement^19,52^. Additionally, the wordsum test has been tested across diverse international samples from countries such as Belgium, Kenya, Nigeria, Singapore, and South Africa [^57–59^].

*Narcissism* was measured using four items from the dirty dozen scale^60^ that has been frequently used to measure narcissism levels in participants^61,62^. Additionally, the measurement of narcissism here reflects trait narcissism and not the clinical diagnosis. The participants are required to rate their level of agreement (1 = strongly disagree to 5 = strongly agree) concerning their personality. Sample items include “I tend to want others to pay attention to me” and “I tend to expect special favors from others.” The response to the four items were averaged to create an index of narcissism.

The variables’ descriptive and reliability details can be found in Tables 1 and 2. All variables met satisfactory levels of reliability, other than for cognitive ability in Indonesia (explained under the sub-section ‘’analysis’’).

**Table 2:** Cronbach’s alpha of all variables under study.

	US	China	Sing	Indo	Malay	Philp	Thai	Viet
Perceived accuracy	.88	.78	.87	.74	.76	.68	.82	.81
Sharing intention	.93	.85	.91	.88	.88	.84	.89	.88
SMF	.92	.87	.93	.87	.85	.86	.87	.86
Cognitive ability	.74	.83	.73	.24	.54	.66	.54	.55
Narcissism	.86	.76	.88	.81	.87	.85	.81	.85

US, United States; Sing, Singapore; Indo, Indonesia; Malay, Malaysia; Philp, Philippines; Thai, Thailand; Viet, Vietnam; SMF, social media fatigue.

### Covariates

This study also includes several covariates in all analytical models to observe the effects of variables of interest over-and-above the core factors that may affect misinformation engagement. The covariates include a) age, b) gender, c) education, d) income, e) political interest, f) social media news use and g) traditional (tv, radio, print) media news use. Other than demographics, political interest and media news use (social and traditional) are included as covariates because they are associated with perceived accuracy and sharing of misinformation. The descriptive details of the covariates are included in Table 1.

### Analytical strategy

At the first step, we conducted ANOVAs and multiple regression analyses for each country to test for preliminary findings. Next, we used the PROCESS macro for SPSS^51^ to test for mediation effects (Model 4) for each country. Finally, we used the same macro to run moderated mediation models with two moderators (Model 76).

Since the reliability for cognitive ability in Indonesia was lower than the satisfactory levels, we excluded this variable from all analytical models in Indonesia. Therefore, while we report on preliminary findings for Indonesia (using ANOVAs and multiple regression analyses), we refrained from running mediation and moderated mediation analyses.

## Results

### Comparing misinformation belief and sharing across countries

**Figure 2:**
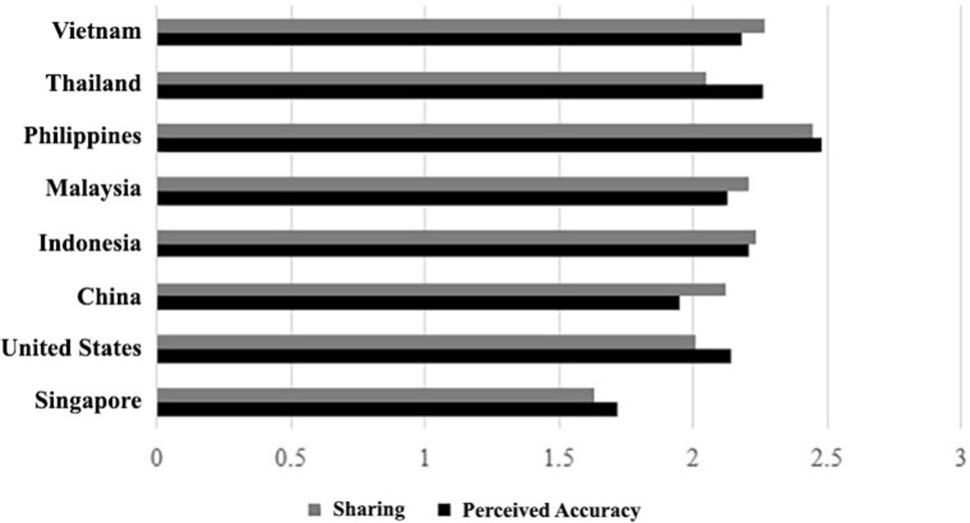
Illustration of perceived accuracy and sharing of misinformation across countries.

Before examining the regression models, we explored whether there were any statistically significant differences between the US, China, Singapore, Indonesia, Malaysia, Thailand, and Vietnam in the perceived accuracy and sharing of COVID-19 misinformation. A one-way ANOVA yielded statistically significant differences between the countries for perceived accuracy, *F*(7,8062) = 58.995, p < 0.001, and sharing *F*(7,8062) = 46.939, p < 0.001, of COVID-19 misinformation. Fisher’s least significant difference (LSD) post hoc tests revealed that the perceived accuracy of COVID-19 misinformation was significantly lower in Singapore compared to the US, China, Indonesia, Malaysia, Thailand, and Vietnam (see Table 3 for more details). The patterns are also illustrated in Fig. 2. In the US, perceived accuracy was significantly higher than in China and Singapore but lower than in the Philippines and Thailand. In China, perceived accuracy was significantly higher than in Singapore but lower than in the US, Indonesia, Malaysia, Thailand, and Vietnam.

**Table 3:** One-way ANOVA coefficients for perceived accuracy across all countries.

Perceived accuracy					
Country	Country	Mean difference	SE	LLCI	ULCI
Singapore	US	− 0.422***	0.041	− 0.503	− 0.342
	China	− 0.232***	0.041	− 0.312	− 0.151
	Indonesia	− 0.485***	0.041	− 0.566	− 0.405
	Malaysia	− 0.409***	0.041	− 0.490	− 0.329
	Philippines	− 0.761***	0.041	− 0.841	− 0.681
	Thailand	− 0.539***	0.041	− 0.619	− 0.458
	Vietnam	− 0.462***	0.041	− 0.542	− 0.381
US	China	0.191***	0.041	0.110	0.271
	Indonesia	− 0.063	0.041	− 0.143	0.018
	Malaysia	0.013	0.041	− 0.068	0.094
	Philippines	− 0.339***	0.041	− 0.419	− 0.258
	Thailand	− 0.116***	0.041	− 0.197	− 0.359
	Vietnam	− 0.039	0.041	− 0.120	− 0.413
China	Indonesia	− 0.254***	0.041	− 0.334	− 0.173
	Malaysia	− 0.178***	0.041	− 0.258	− 0.097
	Philippines	− 0.530***	0.041	− 0.610	− 0.449
	Thailand	− 0.307***	0.041	− 0.388	− 0.227
	Vietnam	− 0.230***	0.041	− 0.310	− 0.150
Indonesia	Malaysia	0.076	0.041	− 0.005	0.157
	Philippines	− 0.276***	0.041	− 0.356	− 0.195
	Thailand	− 0.054	0.041	− 0.134	0.027
	Vietnam	0.024	0.041	− 0.057	0.104
Malaysia	Philippines	− 0.351***	0.041	− 0.432	− 0.271
	Thailand	− 0.129***	0.041	− 0.210	− 0.049
	Vietnam	− 0.052	0.041	− 0.133	0.028
Philippines	Thailand	0.222***	0.041	0.142	0.303
	Vietnam	0.300***	0.041	0.219	0.380
Thailand	Vietnam	0.077	0.041	− 0.003	0.158

Moreover, in Indonesia, perceived accuracy was significantly higher than in Singapore and Malaysia but significantly lower than in the Philippines. In addition, the perceived accuracy in Malaysia was significantly higher than in Singapore and China but significantly lower than in the Philippines and Thailand. On the other hand, the perceived accuracy in the Philippines was significantly higher than in other countries. Similarly, the perceived accuracy in Thailand was significantly higher than in all the countries except Indonesia.

Further LSD post hoc tests revealed that the sharing of COVID-19 misinformation was significantly lower in Singapore compared to the US, China, Indonesia, Malaysia, Thailand, and Vietnam (see Table 4 for more details). In the US, sharing was significantly higher than in Singapore but lower than in all countries except Thailand. In China, sharing was significantly higher than in Singapore and the US but lower than in Indonesia, the Philippines, and Vietnam. Further, in Indonesia, sharing was significantly higher than in Singapore, the US, China, and Thailand but considerably lower than in the Philippines. In addition, the perceived accuracy in Malaysia was significantly higher than in Singapore, US, and Thailand but significantly lower than in the Philippines. Finally, the level of sharing in the Philippines was significantly higher than in all the countries.

**Table 4:** One-way ANOVA coefficients for misinformation sharing across all countries.

Misinformation sharing					
Country	Country	Mean difference	SE	LLCI	ULCI
Singapore	US	− 0.378***	0.049	− 0.474	− 0.281
	China	− 0.489***	0.049	− 0.585	− 0.392
	Indonesia	− 0.599**	0.049	− 0.696	− 0.503
	Malaysia	− 0.575***	0.049	− 0.672	− 0.478
	Philippines	− 0.811***	0.049	− 0.908	− 0.715
	Thailand	− 0.416***	0.049	− 0.514	− 0.320
	Vietnam	− 0.633***	0.049	− 0.730	− 0.536
US	China	− 0.111**	0.049	− 0.207	− 0.014
	Indonesia	− 0.222***	0.049	− 0.318	− 0.125
	Malaysia	− 0.197***	0.050	− 0.294	− 0.101
	Philippines	− 0.433***	0.049	− 0.530	− 0.337
	Thailand	− 0.039	0.049	− 0.136	0.058
	Vietnam	− 0.255***	0.049	− 0.352	− 0.158
China	Indonesia	− 0.111**	0.049	− 0.208	− 0.014
	Malaysia	− 0.087	0.050	− 0.184	0.010
	Philippines	− 0.323***	0.049	− 0.420	− 0.226
	Thailand	0.072	0.049	− 0.025	0.169
	Vietnam	− 0.145***	0.049	− 0.241	− 0.048
Indonesia	Malaysia	0.024	0.050	− 0.073	0.121
	Philippines	− 0.212***	0.049	− 0.309	− 0.115
	Thailand	0.183***	0.049	0.086	0.280
	Vietnam	− 0.034	0.049	− 0.130	0.063
Malaysia	Philippines	− 0.236***	0.050	− 0.333	− 0.139
	Thailand	0.159***	0.050	0.062	0.256
	Vietnam	− 0.058	0.050	− 0.155	0.039
Philippines	Thailand	0.395***	0.049	0.298	0.491
	Vietnam	0.178***	0.049	0.081	0.275
Thailand	Vietnam	− 0.216***	0.049	− 0.313	− 0.120

### Predicting misinformation belief and sharing

In the next step, we ran regression analyses to predict the perceived accuracy and sharing of COVID-19 misinformation. The results suggest that younger people were more likely to perceive COVID-19 misinformation as accurate and share it on social media in all countries except Vietnam (see Tables 5 and 6 for more details). Also, we observed that those with higher political interest were more likely to perceive the false claims as accurate in all countries except China and were more likely to share those claims on social media in most countries.

**Table 5:** Predicting perceived accuracy of misinformation for eight countries.

	US	China	Sing	Indo	Malay	Philp	Thai	Viet
	β	β	β	β	β	β	β	β
Age	− .348***	− .074***	− .236***	− .153***	− .120***	− .172***	− .223***	− .044
Female	− .042	.048	.018	.077*	.020	− .044	.060*	.058
Education	− .080**	− .047	− .024	− .088**	− .131***	− .037	− .064*	− .101***
Income	.056*	.062	− .066*	.064*	− .040	.017	− .041	− .001
Pol interest	.078*	.062	.107***	.107***	.142***	.039***	.135***	.080*
SM news	.108***	.049	.075*	.071*	.014	.104***	− .078*	.043
TV news	− .094**	− .132***	− .034	.005	− .034	− .030	.062	− .097*
Radio news	.201***	.150***	.220***	.105***	.169***	.080	.084***	.130***
Print news	.148***	.195***	.039	.137***	.037	.049	.181***	.252***
$\Delta R$ ^2^	.357***	.106***	.145***	.154***	.088***	.065***	.170***	.133***
SMF	.095***	.120***	.139***	.049	.062***	.080***	.164***	.148***
Cog ability	− .199***	− .209***	− .271***	–	− .222***	− .172***	− .088***	− .124***
Narcissism	.221***	.145***	.169***	.099***	.138***	.117***	.117***	.141***
$\Delta R$ ^2^	.093***	.084***	.150***	.015***	.088***	.051***	.058***	.061***
Total $R$ ^2^	.450***	.190***	.296***	.169***	.175***	.116***	.228***	.194***

US, United States; Sing, Singapore; Indo, Indonesia; Malay, Malaysia; Philp, Philippines; Thai, Thailand; Viet, Vietnam; Pol interest, political interest; SM news, social media news; TV news, television news; SMF, social media fatigue; Cog ability, cognitive ability.

***p < . 001, **p < . 01, *p < . 05.

**Table 6:** Predicting misinformation sharing for eight countries.

	US	China	Sing	Indo	Malay	Philp	Thai	Viet
	β	β	β	β	β	β	β	β
Age	− .331***	− .130***	− .218***	− .175***	− .180***	− .102***	− .245***	− .028
Female	− .066**	.045	− .057	− .004	− .022	− .062*	− .014	− .074*
Education	− .096***	− .004	− .036	− .017	− .140*	− .061	− .049	− .091*
Income	.048	.088***	− .044	.050	− .051	− .020	− .007	− .005
Pol interest	.092***	.122***	.084***	.208***	.197***	.053	.126***	.067
SM news	.137***	.040	.080*	.008	.008	.116***	− .047	.091*
TV news	− .049	− .123***	− .079*	.056	− .080*	.030	.024	− .124*
Radio news	.207***	.135***	.210***	.172***	.195***	.066	.169***	.163***
Print news	.149***	.153***	.085*	.134***	.055	.120**	.120***	.175***
$\Delta R$ ^2^	.380***	.123***	.136***	.244***	.131***	.081***	.175***	.107***
Per Accuracy	.542***	.502***	.618***	.455***	.609***	.387***	.573***	.615***
SMF	.014	− .018	.080***	− .035	.002	.013	− .003	− .054*
Cog ability	− .100***	− .038	− .118***	–	− .062**	− .068***	− .070***	− .133***
Narcissism	.092***	.088***	.065*	.010***	.049*	.098***	.094***	− .019
$\Delta R$ ^2^	.245***	.252***	.442***	.191***	.375***	.173***	.308***	.351***
Total $R$ ^2^	.626***	.375***	.578***	.435***	.506***	.255***	.483***	.459***

US, United States; Sing, Singapore; Indo, Indonesia; Malay, Malaysia; Philp, Philippines; Thai, Thailand; Viet, Vietnam; Pol interest, political interest; SM news, social media news; TV news, television news; SMF, social media fatigue; Cog ability, cognitive ability.

***p < . 001, **p < . 01, *p < . 05.

In addition, those who relied on social media for news were more likely to perceive false claims to be true in the US, Singapore, Indonesia, and the Philippines, while those in Thailand were less likely to do so. Social media news use was also positively related to sharing in the US, Singapore, Philippines, and Vietnam. However, increased reliance on television news negatively predicted the perceived accuracy of COVID-19 misinformation in the US, China, & Vietnam. Similarly, those who were frequent television news consumers were less likely to share misinformation in China, Singapore, Malaysia and Vietnam.

We also observed that those who perceived misinformation about COVID-19 as accurate were more likely to endorse those claims on social media in all countries.

Those with high cognitive ability were less likely to believe and share the claims on social media in most countries. Finally, we observed that while SMF was positively associated with perceiving the misinformation to be accurate (in seven contexts), it was weakly associated with sharing. On the other hand, narcissism was positively associated with perceived accuracy and sharing in all cases other than Vietnam. These relations are further explored in the following section.

### Probing the mechanism of misinformation sharing: role of SMF

Next, we examined how SMF leads to sharing COVID-19 misinformation through perceived accuracy by running mediation analyses using the SPSS PROCESS macro^63^. In all models, SMF was used as the predictor variable, perceived accuracy as the mediator, and sharing as the dependent variable. The bootstrapping method was used to estimate the indirect effects (N = 5000).

**Figure 3:**
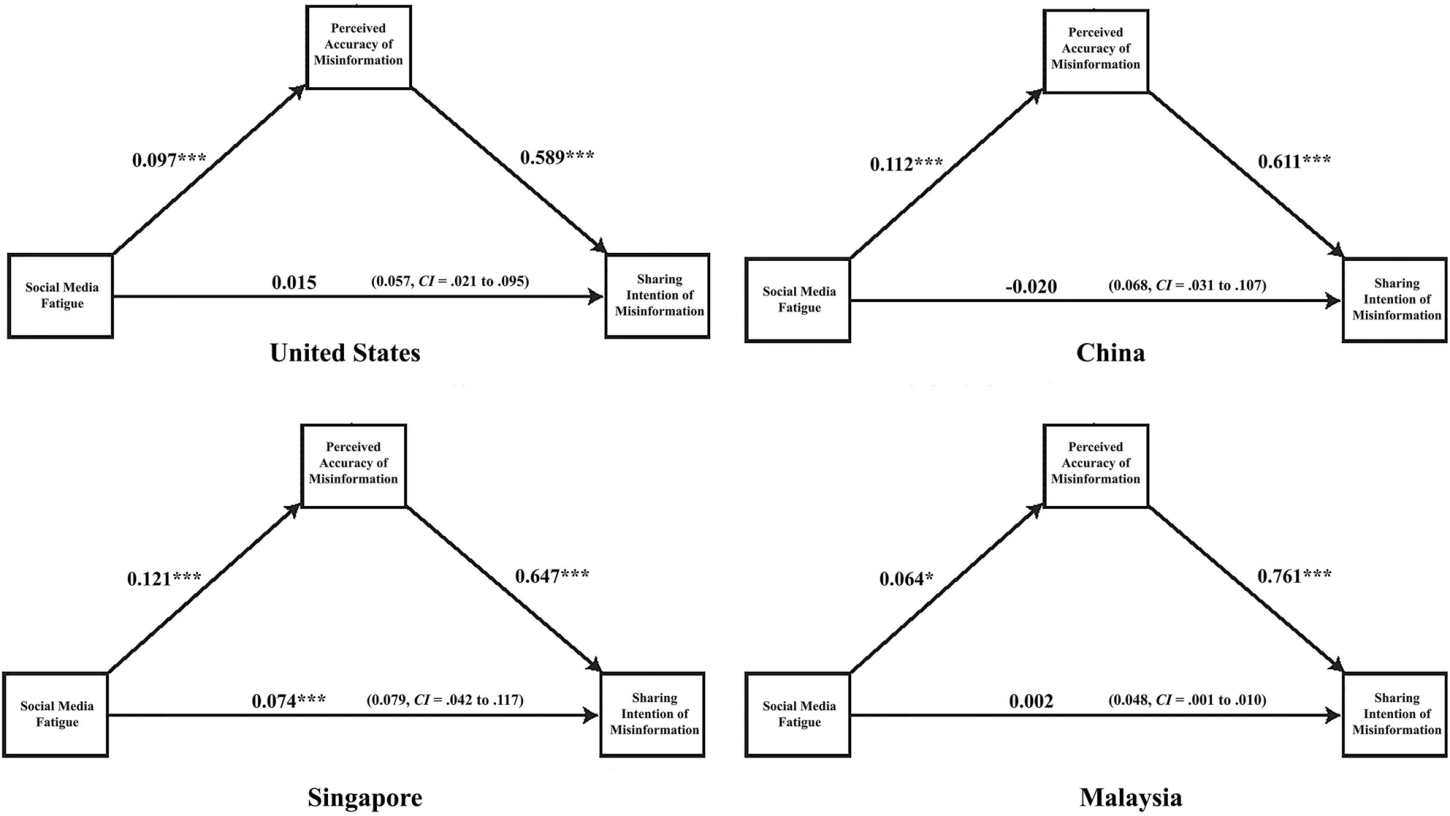
Illustration of the mediation results in the US, China, Singapore and Malaysia. *Note* ***p < .001, **p < .01, *p < .05; Estimates are calculated using the PROCESS macro for SPSS (Model 4; Hayes, 2018). The number in the parenthesis is the indirect effect with LLCI to ULCI. Bootstrap resample = 5000. Statistical controls include *age, gender, education, income, political interest, traditional media and social media news use, narcissism, and cognitive ability*.

**Figure 4:**
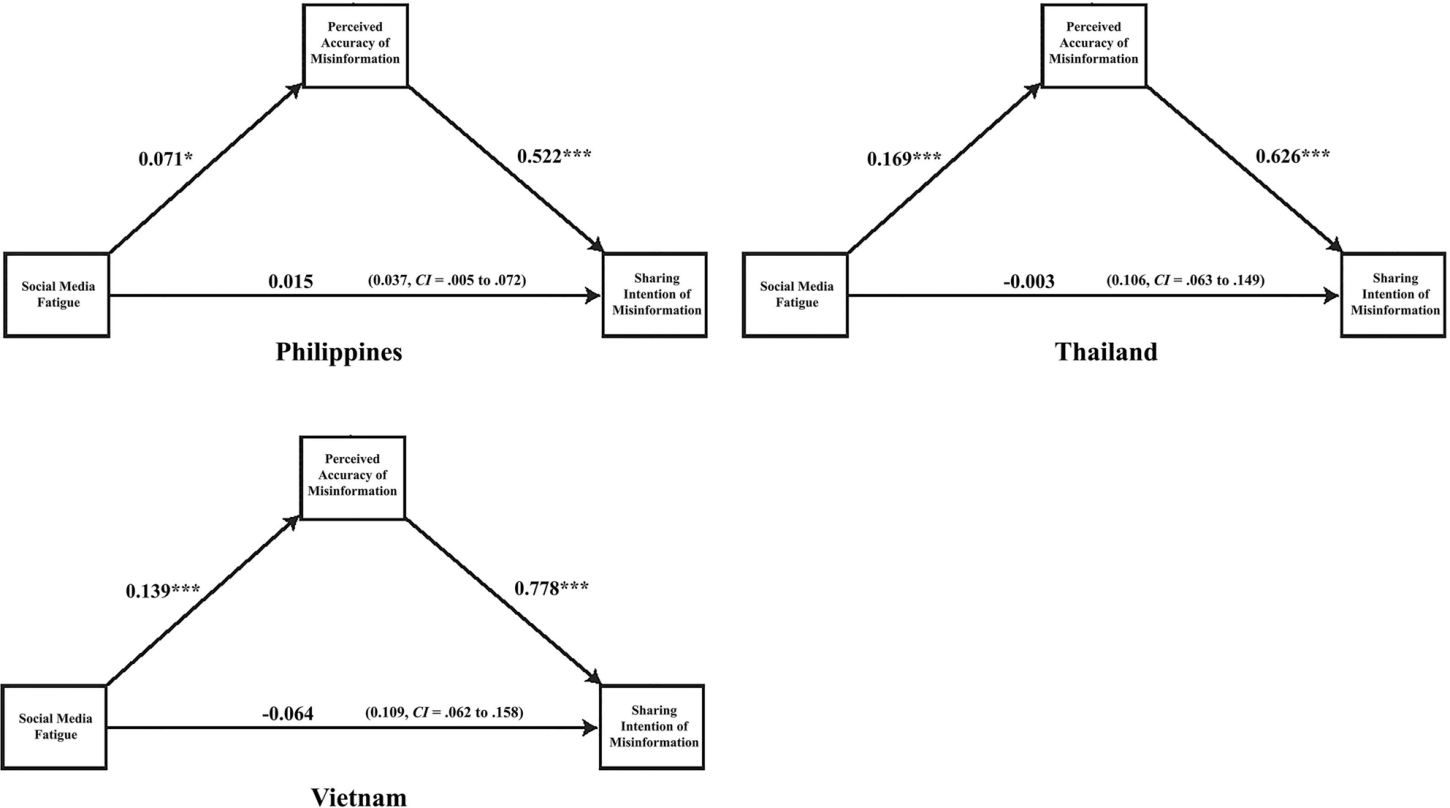
Illustration of the mediation results in Philippines, Thailand and Vietnam. *Note* ***p < .001, **p < .01, *p < .05; Estimates are calculated using the PROCESS macro for SPSS (Model 4; Hayes, 2018). The number in the parenthesis is the indirect effect with LLCI to ULCI. Bootstrap resample = 5000. Statistical controls include *age, gender, education, income, political interest, traditional media and social media news use, narcissism, and cognitive ability*.

The relationships among the variables are plotted in Figs. 3 and 4, and the indirect effects are presented in Table 7. Figures 2 and 3 show that SMF is positively associated with the perceived accuracy of misinformation in all countries. This suggests that those who experience higher levels of social media fatigue are more likely to perceive the misinformation as accurate. Not surprisingly, the perceived accuracy of misinformation is positively associated with sharing intention. These results are consistent across all contexts.

**Table 7:** Mediation results: indirect effects for eight countries.

	Indirect effect				Direct effect			
	Effect	SE	LLCI	ULCI	Effect	SE	LLCI	ULCI
United States	.057***	.019	.021	.095	.015	.025	− .033	.064
China	.068***	.019	.031	.107	− .020	.030	– .079	.040
Singapore	.079***	.019	.042	.117	.074***	.022	.030	.117
Malaysia	.048***	.025	.001	.010	.002	.031	− .059	.064
Philippines	.037***	.017	.005	.072	.015	.035	− .054	.084
Thailand	.106***	.022	.063	.149	− .003	.029	− .059	.053
Vietnam	.109***	.025	.062	.158	− .064*	.030	− .122	− .006

Formal statistical testing of the mediation process (Table 7) suggests that the relationship between SMF and sharing is mediated through the perceived accuracy of misinformation. The lack of direct effects for all contexts, except Singapore, and the consistently significant indirect effects suggest that fatigue experienced by social media users can hinder their processing of misinformation resulting in sharing of such content on social media. Simply put, these results illustrate that SMF translates into sharing false claims related to COVID-19 as individuals perceive such claims to be accurate in all countries examined.

### Probing the mechanism of misinformation sharing: conditional role of cognitive ability and narcissism

Finally, we explored if the effects observed in the mechanism mentioned above are conditional upon individuals’ level of cognitive ability and narcissism. We conducted moderated mediation analyses using the PROCESS macro for SPSS (Model 76). The moderated mediation models included two moderators: cognitive ability and narcissism. The results of the conditional indirect effects for SMF and sharing through perceived accuracy of misinformation at different levels of the moderators (tested at − 1 SD, mean, and + 1SD) are included in Table 8.

**Table 8:** Moderated mediation effects at different levels of two moderators for eight countries.

Cog ability	Narcissism	Effect	Boot SE	Boot LLCI	Boot ULCI
United States					
Low (− 1SD)	Low (1.55)	0.062	0.028	0.008	0.119
	Mean (2.67)	0.107	0.026	0.057	0.158
	High (3.79)	0.160	0.030	0.101	0.219
Mean	Low (1.55)	0.011	0.019	− 0.028	0.049
	Mean (2.67)	0.052	0.017	0.018	0.086
	High (3.79)	0.100	0.028	0.047	0.154
High (+ 1SD)	Low (1.55)	− 0.047	0.021	− 0.091	− 0.008
	Mean (2.67)	− 0.011	0.024	− 0.059	0.034
	High (3.79)	0.034	0.037	− 0.042	0.107
China					
Low (**− **1SD)	Low (2.56)	0.107	0.032	0.048	0.175
	Mean (3.38)	0.108	0.025	0.060	0.159
	High (4.21)	0.109	0.028	0.054	0.166
Mean	Low (2.56)	0.062	0.024	0.016	0.111
	Mean (3.38)	0.065	0.019	0.029	0.102
	High (4.21)	0.067	0.027	0.015	0.123
High (+ 1SD)	Low (2.56)	0.001	0.032	− 0.060	0.063
	Mean (3.38)	0.006	0.029	− 0.053	0.064
	High (4.21)	0.011	0.039	− 0.065	0.088
Singapore					
Low (**− **1SD)	Low (1.70)	0.094	0.036	0.025	0.168
	Mean (2.72)	0.148	0.032	0.090	0.214
	High (3.75)	0.197	0.037	0.129	0.275
Mean	Low (1.70)	0.024	0.024	− 0.022	0.072
	Mean (2.72)	0.084	0.020	0.046	0.124
	High (3.75)	0.138	0.029	0.082	0.197
High (+ 1SD)	Low (1.70)	− 0.051	0.027	− 0.105	0.000
	Mean (2.72)	0.014	0.024	− 0.034	0.061
	High (3.75)	0.073	0.034	0.010	0.142
Malaysia					
Low (− 1SD)	Low (1.80)	0.018	0.043	− 0.064	0.106
	Mean (2.78)	0.063	0.037	− 0.006	0.137
	High (3.75)	0.111	0.040	0.037	0.191
Mean	Low (1.80)	0.007	0.032	− 0.057	0.070
	Mean (2.78)	0.054	0.025	0.004	0.105
	High (3.75)	0.104	0.035	0.038	0.173
High (+ 1SD)	Low (1.80)	− 0.006	0.034	− 0.075	0.059
	Mean (2.78)	0.044	0.033	− 0.020	0.107
	High (3.75)	0.096	0.046	0.008	0.187
Philippines					
Low (− 1SD)	Low (1.96)	0.029	0.020	− 0.008	0.071
	Mean (2.88)	0.047	0.017	0.016	0.083
	High (3.80)	0.066	0.023	0.026	0.115
Mean	Low (1.96)	0.014	0.021	− 0.027	0.056
	Mean (2.88)	0.037	0.016	0.006	0.070
	High (3.80)	0.063	0.026	0.015	0.115
High (+ 1SD)	Low (1.96)	− 0.018	0.034	− 0.084	0.049
	Mean (2.88)	0.011	0.030	− 0.048	0.071
	High (3.80)	0.042	0.040	− 0.036	0.122
Thailand					
Low (− 1SD)	Low (2.30)	0.031	0.039	− 0.050	0.102
	Mean (3.21)	0.110	0.036	0.037	0.178
	High (4.11)	0.193	0.042	0.112	0.275
Mean	Low (2.30)	0.017	0.028	− 0.040	0.068
	Mean (3.21)	0.102	0.022	0.059	0.144
	High (4.11)	0.190	0.032	0.130	0.254
High (+ 1SD)	Low (2.30)	0.002	0.035	− 0.070	0.069
	Mean (3.21)	0.092	0.032	0.028	0.155
	High (4.11)	0.185	0.043	0.105	0.275
Vietnam					
Low (− 1SD)	Low (2.40)	0.025	0.041	− 0.055	0.107
	Mean (3.33)	0.113	0.034	0.052	0.179
	High (4.26)	0.187	0.038	0.117	0.265
Mean	Low (2.40)	− 0.007	0.035	− 0.076	0.059
	Mean (3.33)	0.092	0.024	0.045	0.139
	High (4.26)	0.176	0.031	0.117	0.236
High (+ 1SD)	Low (2.40)	− 0.044	0.044	− 0.138	0.039
	Mean (3.33)	0.065	0.034	− 0.002	0.129
	High (4.26)	0.159	0.039	0.086	0.239

The patterns across all contexts suggested that the strength of the indirect effects, in general, decreases with a decrease in levels of narcissism and an increase in levels of cognitive ability. More precisely, we find weaker indirect effects at higher levels of cognitive ability and either low or average levels of narcissism. Though most importantly, the greatest indirect effects are observed for low cognitive individuals with higher levels of narcissism—these effects are statistically significant for all contexts.

## Discussion

Millions of users rely on social media as an information source to get political, health, and civic news and spend time for entertainment and relational purposes. As such, the observation between SMF and COVID-19 misinformation engagement and its conditional dependencies add value not only to the theoretical discussions surrounding the role of social media on misinformation engagement, but also policymaking focused on restricting the propagation of misinformation. In the last few years, numerous studies have explored public engagement with COVID-19 misinformation, but work on the influence of cognitive and personality traits on user behavior is still nascent. In this study, we used a multi-fold framework focusing on psychological reasoning, personality, and cognitive factors to understand why individuals share misinformation (in this case, COVID-19 misinformation). Our findings offer a cross-national comparison of seven South Asian contexts, benchmarked against the US, a well-studied context for COVID-19 misinformation.

At first, this study finds that respondents in the Philippines were most likely to perceive the misinformation to be accurate and share them on social media. Conversely, those in Singapore were least likely to perceive the misinformation to be accurate and share them. These findings are a reflection of how the public at large perceives misinformation. The Philippines has been riddled with fake news^64^, and information integrity in the country has come under assault^65^. The findings are concerning, given the impact of misinformation on democratic norms within the country^66^. On the contrary, misinformation is not rampant in Singapore, where the government has taken the initiative to provide fact-checking^67^ and has passed laws to limit the propagation of fake news^68^. Moreover, several public campaigns have also been launched to disseminate anti-misinformation educational resources to promote online safety and responsibility^69^.

Beyond the contextual findings, the focus of this study was to identify the mechanisms that lead to COVID-19 misinformation sharing on social media. Here we find that SMF is positively associated with perceiving the misinformation as accurate. These results were consistent across all eight countries. The findings can be explained through several reasons. First, SMF may impair social media users’ critical thinking and increase their confirmation biases. Scholars have argued that the monolithic amount of information that is consumed by individuals on social media can overwhelm the brain, leading to SMF^15,16^. Consequently, individuals are less likely to expend cognitive resources to filter out information^20^, which may lead them to perceive false information as the truth. As such, rather than critically evaluating the accuracy of misinformation, individuals may believe it to be true. The odds of this happening may be more prominent in instances where such misinformation may align with pre-existing biases of users. Secondly, the volume of information available on social media may overwhelm users.

Consequently, this may cause fatigue and make it difficult for users to perform fact-checks. Here, users may accept the misinformation at face value and restrict themselves from ascertaining the credibility of the information. Therefore, instances of looking at fact-checking websites, additional sources, or asking friends or experts who may have the correct knowledge are avoided. Third, the effects of SMF on perceiving the misinformation to be accurate may also be aided by how social media algorithms function by prioritizing controversial, sensational, and emotionally charged content. Finally, being repeatedly exposed to such misinformation may cause individuals to perceive it as accurate. Indeed, recent literature confirms that if users are repeatedly exposed to misinformation on social media, they are more likely to perceive it to be true—a phenomenon termed as illusory truth effect^27,28^—thereby making them more vulnerable to misinformation. Therefore, repeated exposure to misinformation may bolster the perceived accuracy of false information.

The observed direct effects of a strong association between SMF and perceived accuracy of COVID-19 misinformation add rich insights to our understanding of processes driving misinformation belief. Nevertheless, this study also focused on exploring the mechanisms of sharing misinformation. The regression and mediation analyses suggest that SMF is not directly associated with sharing misinformation in most observed contexts. Here, the only positive sign is in Singapore, where those with high SMF are more likely to share misinformation. The indirect effects confirm that across countries, individuals who experience high SMF are more likely to share misinformation on social media because they perceive it to be accurate. Thus, sharing in such cases depends on individuals perceiving the false information to be accurate. These results highlight the need to limit perceptions of misinformation to curb its propagation, especially for groups that are heavy social media users and may experience greater fatigue.

The findings of the direct association between SMF and sharing through perceived accuracy throw light on the processes of misinformation sharing on social media. However, probing this mechanism with attention to the roles of cognitive and personality traits suggests that the observed patterns are not universal and are conditional upon cognitive ability and narcissism.

We find that individuals with high cognitive ability and low or average levels of narcissism are more protected and less likely to share COVID-19 misinformation due to SMF. This may be because of the protection offered by high cognitive abilities, where individuals are less likely to engage with misinformation^35,52^. High cognitive skills enable individuals to analyze information and refrain from misinformation critically. Our findings regarding high cognitive individuals with high levels of narcissism add nuanced insight to the current literature. Unexpectedly, we find that even among high-cognitive-ability groups, individuals with higher narcissistic tendencies are more likely to perceive the misinformation as accurate and share it on social media when they are fatigued. These results indicate that not all high-cognitive-ability individuals would refrain from misinformation engagement and highlight the need to focus on the importance of dark personality traits in misinformation sharing.

Next, we identify that the most extreme indirect effects are observed for low cognitive individuals with high levels of narcissism. This pattern is stable across all eight contexts. We propose the following reasons for this result. First, individuals with low cognitive and high levels of narcissism are more likely to believe misinformation as it may confirm their own biases. Such belief may be a way for them to bolster their ego and sense of self-importance by reaffirming ideas that align with their views. As such, they may be more likely to share such misinformation. Second, with high levels of fatigue, narcissistic individuals could be sharing misinformation as they may be trying to seek attention and gain social influence without applying critical thinking. This is particularly relevant for misinformation which is often characterized by sensational and controversial content eliciting strong emotional reactions from the audience^70–72^. Third, excessive fatigue may amplify impulsivity among low-cognitive narcissists. Narcissists prefer immediate rewards and satisfaction rather than delayed gratification^73^. Thus, it is likely that with high fatigue and limited cognitive ability, narcissists do not make sound judgments about misinformation and share them due to their impulsive nature.

This study also has practical implications for the health of most societies. The adverse effects of social media in impacting mental health^74,75^, incivility^76–78^, and political polarization^79,80^ are widely discussed. This study provides evidence that fatigue arising from excessive use of social media can hamper the judgments of users, which may result in greater misinformation propagation. Therefore, policymakers and social media companies who aim to combat misinformation should pay attention to SMF. Social media companies need to consider SMF while focusing on the designs and affordances of their platforms. Strategies aimed at reducing SMF will not only directly address the issue of fatigue but may also limit misinformation propagation. Similarly, policymakers addressing misinformation propagation should adopt a multi-pronged approach. The efforts should not only be restricted to devising regulations to restrict the spread of misinformation and raising digital literacy, but interventions focused on reducing SMF may help. Besides, the study also supports the need for targeted campaigning among the public. We observe that individuals with certain personality traits and cognition are more susceptible than others. As such, it would be advisable to devise curated strategies targeted at specific groups rather than using a single-lens framework for everyone in society.

It is important here to note that the findings of this study has implications for misinformation, but also for reliable information. We did not measure the effects of narcissism, cognitive ability, and SMF on reliable information. However, it is possible that SMF could increase the perceived accuracy and sharing of reliable information as well. There are no studies which directly examine this possibility, but this serves as an opportunity for future scholarship in this area.

Before we end, it is vital to acknowledge the limitations of this study. First, our analyses are consistent across eight contexts and offer greater generalizability. However, it remains to be seen if these results apply to contexts where social media are not deeply embedded in the information environment. Second, the findings are based on cross-sectional data limiting causal inferences. Nonetheless, the findings are consistent with recent literature, confirming the results’ validity^31,33,35,52^. For instance, existing literature has found that SMF can lead to less deliberation and filtering of information on social media, which may increase individuals’ vulnerability towards misinformation^20^. Further, there is evidence that those who score high on cognitive ability are less likely to believe misinformation^5,29,30^. Third, we analyzed just one aspect of dark personality traits in narcissism, which has been linked with increased engagement with conspiratorial beliefs^40–42^. Yet, it remains to be seen how other characteristics (e.g., psychopathy and Machiavellianism) impact the processes discussed here. Finally, while statistically significant relationships between cognitive ability and various key dimensions (e.g., sharing intention, perceived accuracy, age, and education) across contexts align with existing literature, it is a possibility that the translation of surveys in regional language may have influenced the reliability of cognitive ability. In addition, operationalizing knowledge-based measures through binary responses (0 = incorrect and 1 = correct) presents a challenge in overall reliabilities where scholars are forced to trade validity and reliability^81^. Nevertheless, future studies should replicate the framework with alternate cognitive ability measures (preferably those not based on binary responses).

Notwithstanding the limitation, this study is one of the first to offer cross-national evidence in showing the adverse effects of SMF. It also highlights that any scholarly attempts at understanding public engagement with misinformation must focus on individuals’ personalities and cognitive traits.

## Data Availability

The datasets analysed during the current study are available from the corresponding author on reasonable request.
